# Label-free multimodal nonlinear microscopy enabled by an optical parametric generator

**DOI:** 10.1063/5.0331944

**Published:** 2026-06-18

**Authors:** Alejandro De la Cadena, Edita Aksamitiene, Ruo-Jing Ho, Rohit Bhargava, Stephen A. Boppart

**Affiliations:** 1 Beckman Institute for Advanced Science and Technology, University of Illinois Urbana-Champaign, 405 N. Mathews Avenue, Urbana, Illinois 61801, USA; 2 Department of Bioengineering, University of Illinois Urbana-Champaign, 1406 W. Green Street, Urbana, Illinois 61801, USA; 3 NIH/NIBIB P41 Center for Label-free Imaging and Multiscale Biophotonics (CLIMB), University of Illinois Urbana-Champaign, 405 N. Mathews Avenue, Urbana, Illinois 61801, USA; 4 Department of Electrical and Computer Engineering, University of Illinois Urbana-Champaign, Urbana, Illinois 61801, USA; 5 Cancer Center at Illinois, University of Illinois Urbana-Champaign, Urbana, Illinois 61801, USA; 6 Department of Chemical and Biomolecular Engineering, University of Illinois Urbana-Champaign, Urbana, Illinois 61801, USA; 7 Department of Chemistry, University of Illinois Urbana-Champaign, Urbana, Illinois 61801, USA; 8 Department of Mechanical Science and Engineering, University of Illinois Urbana-Champaign, Urbana, Illinois 61801, USA; 9 Chan Zuckerberg Biohub Chicago, Chicago, Illinois 60642, USA; 10 Interdisciplinary Health Sciences Institute, University of Illinois Urbana-Champaign, Urbana, Illinois 61801, USA

## Abstract

The progress and performance of nonlinear optical microscopy have been paced by breakthroughs in laser technology. Despite substantial advances, a laser source that delivers high peak power and wavelength tunability while supporting multimodal imaging in an experimentally accessible architecture remains elusive. In this contribution, we present a scheme based on an optical parametric generator (OPG). This system bypasses optical cavities and additional amplification stages, providing tunable radiation spanning over 100 nm near the 1500 nm region. We validate the capabilities of the OPG in both nonlinear spectroscopy and microscopy. Spectroscopic performance is established by acquiring coherent anti-Stokes Raman scattering (CARS) spectra of benchmark solvents, followed by narrowband and broadband CARS microscopy of intact and fresh-frozen rodent tissue specimens. In addition to CARS, this light source can also drive two-photon-mediated processes, enabling label-free multimodal imaging, a capability that we demonstrate by co-registering multiphoton autofluorescence, second-harmonic generation, and CARS from freshly excised rodent tissues. Owing to its simple and modular configuration, this design provides an adaptable and cost-effective platform for applications requiring broadband, tunable femtosecond radiation for multimodal nonlinear optical microscopy.

## I. INTRODUCTION

Leveraging nonlinear optical interactions between laser fields and matter, spectroscopic techniques offer a glimpse into the dynamics of fundamental microscopic processes.^1,2^ Using pulsed optical radiation, these approaches reveal the structure and molecular organization of material and biological systems. When coupled with high numerical aperture (NA) objectives, nonlinear optical microscopy converts these interaction-specific signals into image contrast, enabling visualization of specimen composition and morphology at microscopic scales.^3^ As nonlinear optical microscopy provides optical sectioning, reduced photodamage, and multiple complementary intrinsic contrasts,^4^ it has been widely adopted across disciplines, particularly in biomedical imaging.^5–12^

Because these contrasts arise from intensity-dependent light–matter interactions,^13^ the capabilities of nonlinear microscopy are governed by the properties of the excitation source. In particular, nonlinear microscopy benefits from broad spectral bandwidth and wavelength tunability. The former supports ultrashort pulse durations for efficient signal generation, and the latter enables excitation at modality- and sample-specific optimal wavelengths. Consequently, the development and performance of nonlinear microscopy have followed advances in laser technology.

In early nonlinear microscopes, dye lasers were the primary sources, providing high peak power and broad spectral tunability.^14,15^ Although these lasers supported the first demonstrations of nonlinear contrast in biological imaging, the required laser infrastructure was complex and maintenance-intensive. The development of mode-locked solid-state gain media in the 1990s—most notably titanium–sapphire (Ti:sapphire)^16^ and neodymium-doped yttrium aluminum garnet (Nd:YAG)^17^—invigorated nonlinear microscopy by supplying ultrashort (fs and ps) excitation in the near-infrared, a spectral window with reduced tissue scattering and absorption.^18,19^ The advent of diode lasers^20,21^ enabled cheaper and more efficient pump technologies, driving Ti:sapphire- and Nd:YAG-based architectures for nonlinear microscopy for over two decades,^22,23^ albeit at the cost of large and complex systems.

Owing to their ability to deliver high-peak-power femtosecond radiation in compact, turn-key, and cost-effective formats, many contemporary nonlinear microscopes rely on fiber-based^24–27^ or solid-state lasers,^28,29^ e.g., ytterbium (Yb)-based,^30,31^ as excitation sources. Some platforms extend spectral coverage using optical parametric oscillators (OPOs),^32–34^ supercontinuum generation in photonic crystal fibers (PCFs),^35–40^ photonic crystal rods (PC-rods),^41^ or bulk nonlinear crystals.^42–46^ Other sources employ seeded, cascaded optical parametric amplifier (OPA) stages in nonlinear crystals,^47–51^ achieving high peak power and tunability. Collectively, these laser sources have enabled nonlinear microscopy across several contrasts, including second-harmonic generation (SHG),^7,52^ third-harmonic generation (THG),^53,54^ multiphoton absorption fluorescence (MPAF),^55,56^ and coherent Raman scattering—coherent anti-Stokes Raman scattering (CARS),^57^ coherent Stokes Raman scattering (CSRS),^58^ and stimulated Raman scattering (SRS).^59–62^ These technologies have also supported the development of exploratory nonlinear microscopes that rely on less conventional contrasts.^63–67^

Despite these successes, each approach introduces practical constraints. OPOs require optical cavities, increasing system footprint. PCF-based sources can be susceptible to photodamage and may require frequent replacement. PCFs can also exhibit polarization scrambling and higher-order dispersion. Bulk-crystal and PC-rod sources operate at relatively low repetition rates (0.25−5 MHz) to reach the required pulse energy, which can preclude high-speed laser-scanning imaging. Cascaded OPA architectures require precise spatiotemporal overlap across successive stages and additional alignment at the sample plane, which increases footprint and complexity, thus limiting translation. Table I presents the qualitative advantages and limitations of these technologies. Evidently, nonlinear microscopy would benefit from a compact source that does not require additional cavities, a source that not only preserves tunability and peak power but also reduces alignment complexity and cost. Such a source should also support high-speed imaging in conventional laser-scanning to streamline integration.

**TABLE I.** Laser technologies for nonlinear microscopy, presenting qualitative advantages and limitations. Acronyms: Ti:sapphire, titanium:sapphire; Nd:YAG, neodymium-doped yttrium aluminum garnet; Yb, ytterbium; OPO, optical parametric oscillator; PCF, photonic crystal fiber; PC-rod, photonic crystal rod; OPA, optical parametric amplifier; OPG, optical parametric generator.

Laser technology	Advantages	Limitations
Ti:sapphire/Nd:YAG	Broad coverage; benchmark performance	Bulky
Ultrafast fiber/Yb-based solid-state	Compact; turn-key	Limited tunability
OPO	Broad coverage	Bulky
PCF-based supercontinuum	Broad coverage	Prone to photodamage; polarization scrambling; high-order dispersion
PC-rod/bulk-crystal supercontinuum	High pulse energy; broad coverage	Low repetition rate (<2MHz) that leads to low laser-scanning rates
Cascaded OPA stages	Flexible wavelength generation; cavity-free; scalable output	Alignment intensive; increased system complexity
OPG	Cavity-free; continuous tunability; compact; simple implementation	Limited tolerance to high pump power (risk of poling damage); noise degradation; long emission wavelength (impacting spatial resolution)

In this contribution, we present an optical source optimized for label-free multimodal nonlinear microscopy based on a parametric optical generator (OPG), a cavity-free, single-pass device that enables simple implementation, efficient wavelength conversion, broad tunability, high stability, and a compact, cost-effective footprint. We first revisit the operating principle of the OPG, then report the architecture of the source, followed by a thorough characterization of its temporal, spectral, and spatial properties, as well as its noise performance. Next, we establish spectroscopic capability by acquiring CARS spectra of benchmark spectroscopic-grade solvents, followed by narrowband and broadband CARS microscopy of biological samples. Finally, we show that this design can simultaneously drive SHG, MPAF, and CARS, enabling label-free multimodal imaging, as demonstrated using freshly excised rodent tissues. Owing to its simple configuration, we anticipate that this scheme will support applications requiring broadband, tunable femtosecond radiation in a modular, cost-effective platform for biomedical imaging.

## II. THE LASER SOURCE

### A. Optical parametric generator (OPG)

**FIG. 1.**
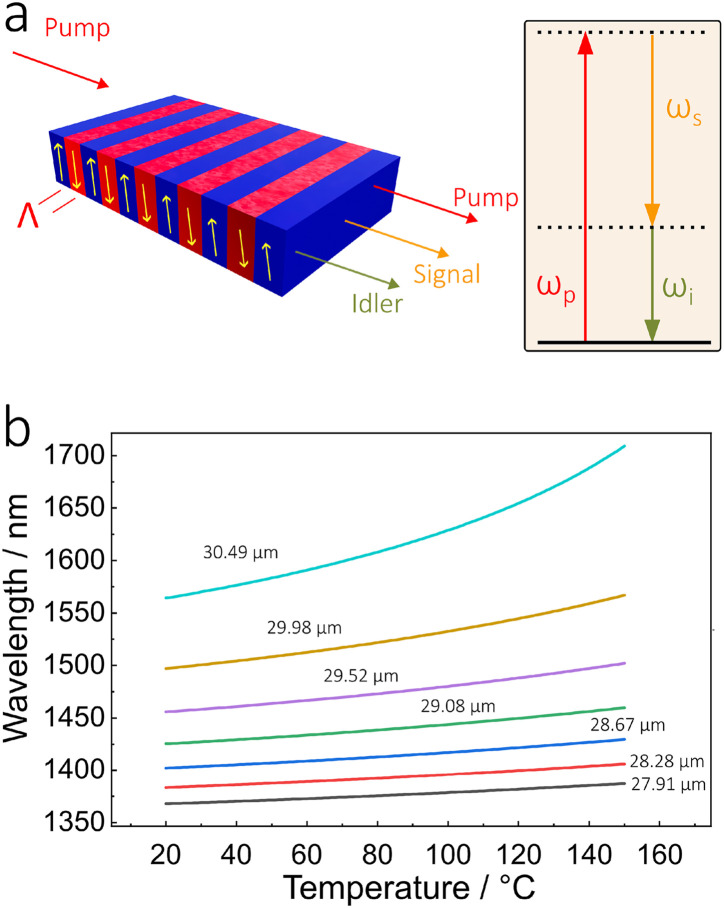
Optical parametric generation in a periodically poled crystal. (a) Schematic of a PPLN crystal with poling period Λ; arrows indicate the orientation of the ferroelectric domains. The inset illustrates energy conservation in OPG, where a pump photon at *ω*_*p*_ is converted into a signal photon at *ω*_*s*_ and an idler photon at *ω*_*i*_, satisfying *ω*_*p*_ = *ω*_*s*_ + *ω*_*i*_, with *ω*_*p*_ > *ω*_*s*_ > *ω*_*i*_. (b) Calculated signal wavelength *λ*_*s*_ as a function of temperature for several poling periods Λ obtained by numerically solving the first-order quasi-phase-matching condition Δ*k* = 0 at a fixed pump wavelength *λ*_*p*_ = 1030 nm. The selected Λ values and *λ*_*p*_ correspond to commercially available PPLN crystals and Yb-based laser sources.

An OPG is an optical device that can generate tunable, coherent radiation in the near-infrared and mid-infrared ranges.^68–71^ Because an OPG operates in a single pass, it does not require a cavity. Thus, it offers a simple, flexible, and robust architecture with a compact footprint. The OPG is based on second-order nonlinear materials that support periodic poling,^72^ such as lithium niobate (PPLN). Since lithium niobate is ferroelectric, the sign of its nonlinear coefficient can be reversed by periodic domain inversion,^73,74^ creating a grating with spatial period Λ, Fig. 1(a). This grating supplies an additional wave vector *K*_QPM_ that compensates the phase mismatch between the interacting waves, enabling parametric frequency conversion. Thus, for a fixed pump wavelength *λ*_*p*_ and temperature *T*, the poling period Λ provides direct control over the emitted signal *λ*_*s*_ and idler *λ*_*i*_ wavelengths. This control grants access to wavelength combinations that are difficult or impossible to attain using birefringent phase matching.^75^

The output wavelengths of an OPG obey energy conservation,

$$\frac{1}{\lambda_{p}}=\frac{1}{\lambda_{s}}+\frac{1}{\lambda_{i}},$$ (1)

while QPM requires the wave vector mismatch Δ*k* to vanish,

$$\begin{array}{ll} \Delta k & =k_{p}\left(\lambda_{p},T\right)-k_{s}\left(\lambda_{s},T\right)-k_{i}\left(\lambda_{i},T\right)-K_{\text{QPM}}\\ & =2\pi \left(\frac{n_{p}\left(\lambda_{p},T\right)}{\lambda_{p}}-\frac{n_{s}\left(\lambda_{s},T\right)}{\lambda_{s}}-\frac{n_{i}\left(\lambda_{i},T\right)}{\lambda_{i}}-\frac{m}{\Lambda }\right)=0, \end{array}$$ (2)

where *k*_*j*_(*λ*_*j*_, *T*) = 2*πn*_*j*_(*λ*_*j*_, *T*)/*λ*_*j*_ is the wave number (propagation constant) and *n*_*j*_(*λ*_*j*_, *T*) is the refractive index for *j* ∈ {*p*, *s*, *i*}, while *K*_QPM_ = 2*πm*/Λ, with *m* being the QPM order (*m* = 1 corresponds to first-order QPM). Figure 1(b) presents the calculated signal tuning *λ*_*s*_(*T*), obtained by numerically solving the coupled energy conservation [Eq. (1)] and QPM [Eq. (2)] relations. Both the poling period Λ and the temperature *T* provide wavelength control, with Λ enabling coarse adjustment and *T* fine-tuning.

### B. Architecture of the laser source

The OPG enables selection of the signal–pump frequency separation, allowing wavelength combinations that support simultaneous multiphoton and vibrational contrasts.^76^ A 1030 nm pump is both readily available and efficient for driving MPAF and SHG, while a signal wavelength near 1500 nm provides a pump–signal detuning that can coherently excite C–H stretching vibrations. Thus, by selecting an appropriate Λ and *T*, the OPG offers a strategy for co-registering structural (SHG), functional (MPAF), and vibrational (CARS) contrasts.

**FIG. 2.**
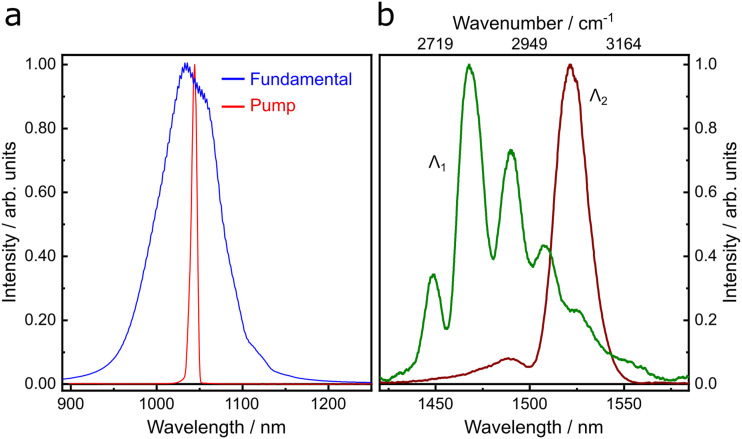
Spectra of the optical radiation from this source. (a) Fundamental (blue) and narrowband pump (red). (b) Signal (Stokes) spectral profiles in the wavelength domain (bottom axis) for different PPLN poling periods (Λ_1_ = 28.67 *μ*m and Λ_2_ = 30.49 *μ*m). The top axis shows the vibrational modes driven by the pump–Stokes detuning.

As primary source, we use a custom-made Yb-based solid-state laser (Flint, Light Conversion) operating at a 10 MHz repetition rate and delivering 75 fs pulses. The laser provides up to 5 W of average power, which is split into two arms to produce a pair of beams centered at 1030 nm, fundamental 1 and fundamental 2 (blue curve in Fig. 2(a). Both outputs originate from the same Yb-based oscillator and are delivered as two spatially separated but synchronized beams.

The Stokes radiation is supplied by the signal beam of the OPG. To obtain it, a 1.0 W fraction of fundamental 2 is focused with a 150 mm lens into a 10 mm-long MgO-doped periodically poled lithium niobate crystal (MgO:PPLN; Covesion Ltd.). The crystal contains nine adjacent gratings with poling periods spanning Λ = 27.91–31.59 *μ*m, enabling signal generation near 1500 nm. Figure 2(b) shows representative spectra obtained using Λ_1_ = 28.67 *μ*m and Λ_2_ = 30.49 *μ*m. Different poling periods are better suited for targeting distinct vibrational sub-bands; for example, Λ_1_ most efficiently accesses Raman shifts near 2820 cm^−1^, whereas Λ_2_ provides access to higher Raman shifts within the C–H region. The PPLN is temperature-stabilized at 40 °C in an oven, mainly to improve long-term stability and reduce crystal degradation. Residual fundamental 2 light, together with spurious visible emission generated in the crystal, is removed using a long-pass filter. The resulting signal is not only consistent with Eqs. (1) and (2) but also exhibits excellent long-term stability. In particular, a maximum conversion efficiency of ∼25% was obtained using the 10-mm-long MgO:PPLN crystal with Λ = 27.91 *μ*m at 40 °C, pumped with 1.0 W of fundamental 2. Under these conditions, the generated signal spanned a bandwidth of up to ∼100 nm.

Since the spectral resolution in CARS obeys the bandwidth of the pump,^77^ a total of 0.25 W of fundamental 1 is sent to a spectral shaper to produce a narrowband beam. The spectral shaper (inset in Fig. 5) is arranged in a 4f configuration, following the design by Liberale and colleagues.^59^ In particular, we used a blaze grating (GR25-1210, 1200 grooves/mm, Thorlabs) to disperse fundamental 1, a 120 mm lens conjugated to the grating, and a slit to clip all but a narrow portion of fundamental 1. The narrowband beam is reflected back to be spatially combined and used as the pump in the experiments, red curve in Fig. 2(a).

By closing the slit, a higher spectral resolution in the CARS contrast can be achieved. However, this improvement comes at the expense of pump power and pulse width, which particularly impacts the MPAF contrast. Thus, for the CARS-only experiments (Secs. IV C 1, IV C 2, and IV C 3), the slit was closed to achieve an estimated spectral resolution of ∼20 cm^−1^, whereas for multimodal imaging (Sec. V), the slit was opened slightly to maintain sufficient MPAF contrast. The resolution stated was estimated from the time–bandwidth product of the narrowband pump. Using the measured autocorrelation width of *τ*_AC_ ≈ 1.33 ps and assuming a Gaussian pulse, the corresponding pulse duration is *τ* ≈ 0.94 ps. This gives a transform-limited bandwidth of $\Delta \tilde{\nu }\approx 0.44/\left(c\tau \right)\approx 16$ cm^−1^, which we report as an approximate spectral resolution of ∼20 cm^−1^.

Another peculiarity of this source is its 10 MHz repetition rate, which is relatively low compared with the 40–80 MHz repetition rates typically used in nonlinear microscopes. We recognize three advantages of this configuration. (i) For a fixed pulse width and fixed average power deposited into the sample, a lower repetition rate increases the pulse energy and, consequently, the peak power of the femtosecond pulses. This allows nonlinear effects to be driven more efficiently in both the specimens and nonlinear crystals. (ii) A longer interpulse period provides more time for energy dissipation between pulses, which can help mitigate thermal loading in the specimen. At the same average power, a low repetition rate may reduce heat accumulation relative to higher-repetition-rate excitation, potentially reducing phototoxic effects associated with the high pulse energies required in multiphoton microscopy.^78^ (iii) A 10 MHz synchronization signal from the laser is easier to integrate with synchronization and instrumentation hardware than equivalent 40–80 MHz signals. This integration bypasses clock dividers or frequency-conversion electronics.

For CARS imaging alone, a 10 MHz repetition rate supports standard galvo-scanning rates, including pixel dwell times below 10 *μ*s. Using modulation-transfer detection, we obtained images of microplastics with time constants as short as 0.1 *μ*s, which corresponds to the 100 ns interpulse period of the 10 MHz source, i.e., approximately one pulse within that time window (Fig. 1a of the supplementary material). In this work, the laser source was optimized primarily for CARS imaging, and the generation of multiphoton contrasts was partially compromised. However, when the pulses are optimized for multiphoton excitation by maintaining ultrashort pulse durations, these contrasts can also be efficiently driven at a 10 MHz repetition rate (Figs. 1b and 1c of the supplementary material), making this low rep-rate fully compatible with practical label-free imaging.

Finally, the current benchtop prototype of this source occupies ∼0.75 × 0.47 × 0.15 m^3^ on the optical table, with a separate Yb oscillator measuring 0.43 × 0.19 × 0.15 m^3^. The total cost of the laser and associated components is approximately USD 80k. For comparison, representative commercial coherent Raman scattering (CRS) sources typically occupy footprints of 1.08 × 0.50 × 0.19 m^3^ or 0.95 × 0.36 × 0.47 m^3^ and are commonly priced in the range of USD 280–350k. With further engineering and enclosure, the footprint of the present benchtop configuration is expected to be reduced; based on the current layout, a packaged implementation is projected to be ∼45% smaller (footprint area) than representative commercial CRS platforms, facilitating integration.

### C. Temporal and spectral characterization of the OPG signal pulses

**FIG. 3.**
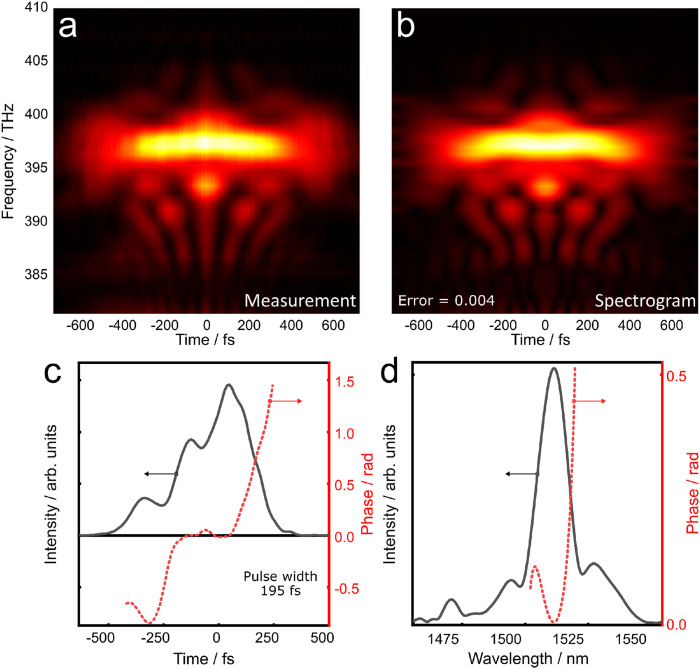
SHG-FROG characterization of the signal (Stokes) pulses (Λ_2_ = 30.49 *μ*m). (a) Measured SHG-FROG trace. (b) Retrieved SHG-FROG spectrogram. (c) Retrieved temporal intensity and phase. (d) Retrieved spectral intensity and phase.

To characterize the signal (Stokes) pulses from the OPG, we retrieved its temporal and spectral intensity and phase using second-harmonic-generation frequency-resolved optical gating (SHG-FROG).^79,80^ Figures 3(a) and 3(b) show the experimental and retrieved SHG-FROG maps for the signal pulses, while Figs. 3(c) and 3(d) report the retrieved temporal and spectral intensity and phase profiles. Note that the signal beam exhibits a relatively small but linear spectral phase, implying that these pulses experienced a time delay with minimal temporal broadening. This observation is consistent with the fact that the optical elements following the PPLN have either negligible or negative group-velocity dispersion at 1520 nm, thus introducing close to zero (or even negative) group-delay dispersion (GDD), leaving the signal radiation with a pulse duration of 195 fs. Because the signal pulses are an order of magnitude shorter than the pump pulses, the pump acts as a narrowband probe, and we, therefore, expect CARS spectra with near-Raman line shapes. This set of measurements benchmarks the stability, coherence, and reproducibility of the PPLN radiation.

### D. Spatial profile of the OPG signal

**FIG. 4.**
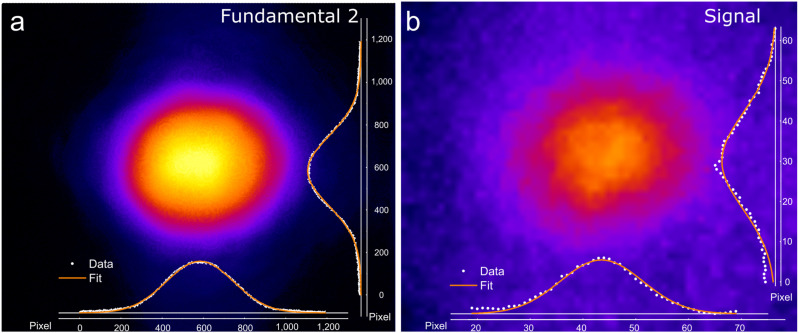
Beam profile of the OPG. (a) Beam profile of fundamental 2. (b) Beam profile of the signal (Stokes) beam. The insets display intensity profiles taken along the central horizontal and vertical axes of the intensity distributions.

A spatial distribution with a Gaussian mode is desirable in both pump and signal beams.^81^ This distribution leads to optimal focusing, high spatial coherence, predictable propagation, and ultimately efficient nonlinear signal generation. Figure 4(a) shows the beam profile of fundamental 2 before the PPLN. The beam of the solid-state laser is Gaussian. However, the beam emerging from the PPLN crystal, i.e., the signal beam, also maintains a Gaussian profile [Fig. 4(b)]. The Gaussian profile present in both pump and signal beams confirms the suitability of this design for nonlinear microscopy.

## III. THE IMAGING SYSTEM

**FIG. 5.**
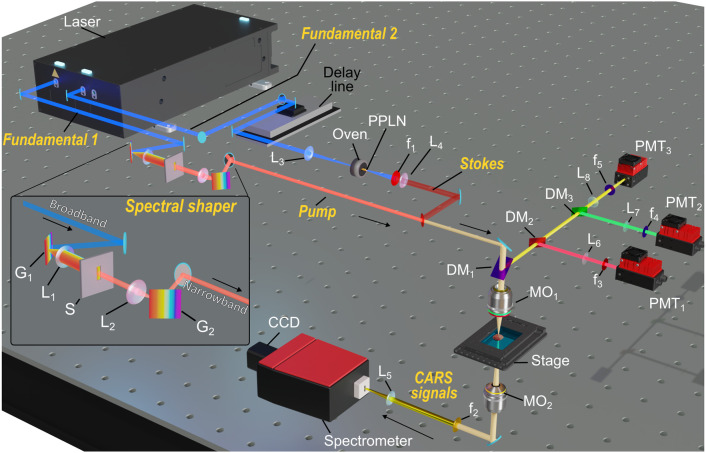
Schematic of the system. The inset shows a zoom view of the spectral shaper (depicted in transmission mode for the sake of clarity). L: Lens. f: Filter. PPLN: magnesium oxide-doped periodically poled lithium niobate. MO: Microscope objective. DM: Dichroic mirror. G: Grating. S: Slit. PMT: Photomultiplier tube. The spectrometer captures the forward CARS, while PMT_1_, PMT_2_, and PMT_3_ detect the epi-CARS, epi-SHG, and epi-MPAF, respectively.

A schematic of the system is shown in Fig. 5. The pump and signal (Stokes) beams were temporally matched with a motorized delay line (X-LSM200A, Zaber) and spatially combined using a dichroic mirror (FF01-1326/SP-25, Semrock), which directs the beams to the subsequent experiments. The sample was raster scanned with a translation stage (FTP-2000, ASI) and imaged with a pair of microscope objectives: a driving objective (CFI60, NA = 0.80, Nikon) and a collection objective (Plan-Apochromat 63× Oil DIC M27, NA = 1.4, Carl Zeiss).

The forward-CARS signal was dispersed by a spectrograph (IsoPlane 160, Princeton Instruments) equipped with a 1200 grooves/mm grating and recorded with a camera (ProEM-HS: 512BX3, Princeton Instruments). A bandpass filter (FL-004520, Semrock) rejected the pump, Stokes, and other nonlinear signals (e.g., autofluorescence, second- and third-harmonic generation) while transmitting the CARS signal within 750–850 nm.

Nonlinear signals for multimodal imaging were collected in the epi direction while the sample was raster scanned with a galvanometer (GVS012, Thorlabs) and separated from the excitation using a dichroic mirror (FF875-Di01, Semrock). The CARS band was extracted from the remaining nonlinear signals with a dichroic beam splitter (Di02-R785, Semrock); a collection lens and a bandpass filter (FF02-809/81-25, Semrock) were placed before the CARS detector. A short-pass filter (cutoff ≈780 nm) transmitted SHG and MPAF. SHG was isolated with a dichroic (Di02-R532, Semrock) and a bandpass filter (FF01-509/22, Semrock), while MPAF was selected with a bandpass filter (FF01-559/34, Semrock), capturing fluorescence in the 540–580 nm range.

The epi CARS was detected with an analog-output photomultiplier tube (AO-PMT; H16722-50, Hamamatsu), and the signal was routed to a transimpedance amplifier (TIA60, Thorlabs). SHG was detected with a second AO-PMT (H16722-40, Hamamatsu) and TIA (TIA60, Thorlabs). Both the CARS and SHG channels were demodulated simultaneously by a multichannel lock-in amplifier (Moku:Pro, Liquid Instruments). The MPAF signal was detected with a photon-counting PMT (PC-PMT; H16721-40, Hamamatsu) to maximize sensitivity. The multimodal microscope was controlled by an in-house LabVIEW program that synchronized readout using two DAQ boards (PCI-6323, National Instruments).

For microscopy experiments, the pump power was set to 6 mW at the sample plane while that of the Stokes to 25 mW. This choice was made to minimize pump-induced photodamage while maintaining adequate signal-to-noise ratio (SNR).

## IV. EVALUATION OF THE OPG SOURCE FOR CARS MICROSPECTROSCOPY

CARS requires the superposition of two fields—the pump *E*(*ω*_pu_) and the Stokes *E*(*ω*_*S*_)—to drive a vibrational coherence.^82–84^ This requirement makes CARS a direct validation of the OPG output for coherent nonlinear excitation. In this section, we evaluate the OPG source in the context of CARS microspectroscopy. We present its noise performance and the contribution of relative intensity noise to CARS detection (Sec. IV A). We then discuss the implications of using a long Stokes wavelength for CARS imaging in terms of spatial resolution (Sec. IV B 1) and penetration depth (Sec. IV B 2). Finally, we present CARS spectroscopic and imaging results, including spectra of benchmark solvents (Sec. IV C 1), narrowband label-free imaging of freshly excised rodent tissues (Sec. IV C 2), and broadband CARS microscopy for label-free histological visualization (Sec. IV C 3).

### A. Noise performance of the OPG in the context of CARS microscopy

We investigated the noise performance of the OPG by measuring its relative intensity noise (RIN). The RIN evaluates laser noise at a given modulation frequency *f* and is defined as the power noise *δ*(*f*) normalized by its mean power $\bar{P}$. A lower RIN indicates more stable, or “quiescent,” pulses, which are essential for high-sensitivity applications.^85,86^

**FIG. 6.**
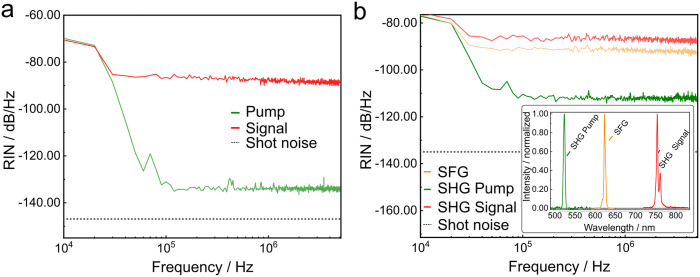
Noise performance of the optical source. (a) Relative intensity noise (RIN) of the pump (green) and signal (Stokes, red) beams. The dotted curve corresponds to the shot noise of the experiments. (b) RIN of second-order nonlinear optical processes driven by the pump (green), signal (Stokes, red), and their sum-frequency generation (orange). The dotted line depicts the shot noise of these experiments. The inset shows the spectra of the *χ*^(2)^ processes.

The RIN of the pump and signal (Stokes) beams is presented in Fig.  6(a). In all RIN measurements, the shot noise of the different beams was kept equal to control for the spectral sensitivity of the photodiode. Figure 6(a) shows that the RIN of the pump beam was satisfactorily low. However, the light emerging from the PPLN exhibited a substantial noise increase. Noise degradation in quasi-phase-matching has been attributed to a combination of spontaneous Raman scattering, thermal effects, and parasitic nonlinear optical processes generated inside the PPLN.^87–90^

We also studied the impact of these fluctuations on nonlinear optical processes generated by the OPG. We measured the RIN of radiation generated by second-order interactions of the pump and Stokes beams in *χ*^(2)^ crystals. Figure 6(b) shows the RIN of the SHG of the pump and signal (Stokes) beams, along with their sum-frequency generation (SFG). The inset displays the corresponding spectra for reference. The pattern observed in the RIN of the fundamental beams was mirrored in their second-order nonlinear signals: the SHG of the pump remained low, while the SHG of the signal (Stokes) was higher. Notably, the RIN of the SFG—a nonlinear process driven by both beams—was higher than the SHG of the pump but lower than the SHG of the signal (Stokes).

To investigate the impact of the RIN on the CARS readout, we performed a two-part analysis. The first part pertains to the experimental determination of the mean CARS voltage readout, ${\bar{V}}_{\text{CARS}}$, and the absolute CARS voltage variance, $\sigma_{V,CARS}^{2}$, from fixed-point acquisitions obtained at a given lock-in amplifier (LIA) time constant, *τ*. The second part deals with the estimation of the equivalent CARS voltage variance expected from the RIN of the pump and Stokes beams.

For the experimental part, we used glycerol because it does not evaporate easily and produces strong CARS in the C–H stretching. The CARS signal was acquired at a fixed sample position, i.e., without sample scanning, using an AO-PMT routed to a LIA demodulating at 10 MHz. Because each pixel in a frame corresponds to a bandwidth-limited readout, we calculated the variance across all pixels within each individual frame. These measurements were performed with excitation ON and excitation OFF, as shown in Fig. 2 of the supplementary material. The excitation-on voltage variance, $\sigma_{V,on}^{2}$, and the excitation-off voltage variance, $\sigma_{V,o\mathrm{ff}}^{2}$, were each defined as the mean of the corresponding per-frame variances across sequential acquisitions. The CARS voltage variance was then calculated as $\sigma_{V,CARS}^{2}=\sigma_{V,on}^{2}-\sigma_{V,o\mathrm{ff}}^{2}$.

To estimate the RIN contribution, we used the definition of the RIN as the power spectral density of *fractional* intensity fluctuations.^91,92^ The RIN allowed us to calculate the fractional intensity variance, $\sigma_{\text{frac},i}^{2}$, of beam *i* over an effective detection bandwidth *BW*. Since the RIN values in Fig. 6 are in dB/Hz, they were converted to linear units, giving $\sigma_{\text{frac},i}^{2}\approx 10^{{\text{RIN}}_{i}/10}\times BW$. Importantly, we assumed a constant RIN around the modulation frequency. From the pump and Stokes RIN values, we obtained $\sigma_{\text{frac},pu}^{2}$ and $\sigma_{\text{frac},S}^{2}$, respectively. Because the CARS signal scales as $I_{\text{CARS}}\propto I_{\text{pu}}^{2}I_{S}$, pump fluctuations contribute $4\sigma_{\text{frac},pu}^{2}$ to the fractional CARS variance, whereas Stokes fluctuations contribute $\sigma_{\text{frac},S}^{2}$. Assuming uncorrelated pump and Stokes fluctuations, the combined RIN-induced fractional CARS variance is, therefore, $\sigma_{\text{frac},CARS}^{2}=4\sigma_{\text{frac},pu}^{2}+\sigma_{\text{frac},S}^{2}$. To compare with the experimental result, this fractional variance was converted to an equivalent absolute CARS voltage variance by $\sigma_{V,CARS,RIN}^{2}={\bar{V}}_{\text{CARS}}^{2}\sigma_{\text{frac},CARS}^{2}$. The results are summarized in Table II.

**TABLE II.** Comparison between the measured and RIN-estimated contributions to the CARS voltage variance for a lock-in amplifier demodulating at 10 MHz with a time constant of *τ* = 20 *μ*s, using 6.8 mM glycerol, 15 mW Stokes power, and 10 mW pump power. The voltage variances under excitation-ON and excitation-OFF conditions were calculated from 62 400 to 47 400 fixed-point measurements, respectively. The RIN contribution was estimated from the measured RIN spectra using an effective detection bandwidth of *BW* = 1/(4*τ*) = 12.5 kHz.

Source	RIN	Equivalent CARS variance
	(dB/Hz)	$\left(V_{\text{rms}}^{2}\right)$
Pump contribution, ${\bar{V}}_{\text{CARS}}^{2}\times \sigma_{\text{frac},pu}^{2}$	−135	1.38 × 10^−14^
Pump contribution in CARS, ${\bar{V}}_{\text{CARS}}^{2}\times 4\sigma_{\text{frac},pu}^{2}$	−135	5.53 × 10^−14^
Stokes contribution, ${\bar{V}}_{\text{CARS}}^{2}\times \sigma_{\text{frac},S}^{2}$	−85	1.38 × 10^−9^
Combined RIN contribution, ${\bar{V}}_{\text{CARS}}^{2}\times \left(4\sigma_{\text{frac},pu}^{2}+\sigma_{\text{frac},S}^{2}\right)$	⋯	**1.38 × 10** ^**−9**^
Measured LIA variance, excitation ON, $\sigma_{V,on}^{2}$	⋯	**4.12 × 10** ^**−8**^
Measured LIA variance, excitation OFF, $\sigma_{V,o\mathrm{ff}}^{2}$	⋯	1.79 × 10^−8^
CARS variance, $\sigma_{V,CARS}^{2}=\sigma_{V,on}^{2}-\sigma_{V,o\mathrm{ff}}^{2}$	⋯	**2.33 × 10** ^**−8**^

The CARS voltage variance $\sigma_{V,CARS}^{2}$ was ∼17× larger than the combined RIN-only estimate of $1.38\times 10^{-9}\;V_{\text{rms}}^{2}$. This finding indicates that the RIN provides a measurable but non-dominant contribution to the CARS readout noise under our experimental conditions, accounting for ∼5.9% of the CARS variance. The residual variance likely contains additional excitation-dependent and signal-dependent noise contributions, including PMT shot and excess noise, analog-output and mechanical fluctuations, and sample-dependent variations.

Although the RIN was not the dominant noise source in our CARS measurements, it may have different implications for SRS, which is arguably the more impactful coherent Raman technique for quantitative vibrational imaging.^93–95^ In SRS, the detected signal is a small intensity modulation superimposed on an intense beam. Therefore, elevated RIN in the Stokes beam can affect stimulated Raman gain (SRG), in which the Stokes beam is detected, thus limiting fast SRS imaging. A viable strategy for deploying the OPG in SRS is stimulated Raman loss (SRL), in which the Stokes beam is kept narrowband and modulated while the pump is detected. By measuring the pump instead of the noisier Stokes, this configuration may reduce the impact of the RIN in the Stokes delivered by the OPG. However, a definitive assessment of SRS feasibility with the OPG requires SRG/SRL measurements.

### B. Implications of a long-Stokes wavelength in CARS imaging

Conventional CARS microscopes and CRS implementations, in general,^43,82,96,97^ typically operate with pump wavelengths near *λ*_*pu*_ ∼ 790 nm and Stokes wavelengths in the range *λ*_*S*_ ∼ 1030–1100 nm. In this context, the Stokes beam produced by the OPG, centered at *λ*_*S*_ ∼ 1490 nm, is unusual. Although light in this spectral range lies farther from electronic resonances and may reduce tissue photodamage, its longer wavelength impacts the spatial resolution and imaging depth.

#### 1. Spatial resolution

To quantify the spatial-resolution penalty introduced by a Stokes beam centered near ∼1490nm, we simulated the effective CARS generation point-spread function (PSF). We first computed the pump and Stokes field distributions in the focal volume of a microscope objective (NA = 0.8) for a conventional configuration (case 1, *λ*_*pu*_ = 786 nm, *λ*_*S*_ = 1030 nm) and for the OPG-based approach (case 2, *λ*_*pu*_ = 1030 nm, *λ*_*S*_ = 1490 nm). Both cases target a vibrational mode at 3000 cm^−1^. The focused pump and Stokes fields were obtained by following the formalism introduced by Richards and Wolf,^98^ while the effective CARS response was defined^99^ as $I_{\text{CARS}}\propto I_{pu}^{2}I_{S}\propto {\left|E_{pu}^{2}E_{S}^{*}\right|}^{2}=|E_{pu}{|}^{4}|E_{S}{|}^{2}$.

**FIG. 7.**
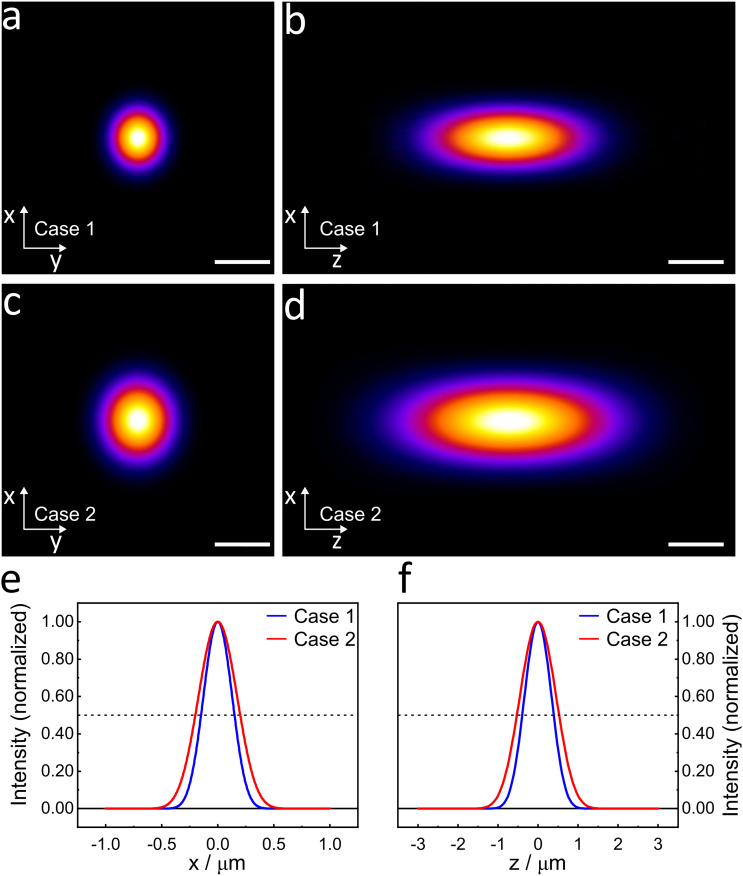
Simulated CARS excitation PSFs, computed as $I_{\text{CARS}}\propto |E_{p}^{2}E_{s}^{*}{|}^{2}=|E_{p}{|}^{4}|E_{s}{|}^{2}$, using a Richards and Wolf formalism with a microscope objective of NA = 0.8. Here, *x* and *y* are lateral coordinates, and *z* is the axial coordinate. The excitation beams propagate along the *z* direction. The simulation assumes pump and Stokes linearly polarized in *x*. Panels (a) and (b) show normalized sections of the CARS PSF in the *xy* and *xz* planes, respectively, for case 1 using *λ*_*pu*_ = 786.9 nm and *λ*_*S*_ = 1030 nm. Similarly, panels (c) and (d) show the corresponding sections for case 2 using *λ*_*pu*_ = 1030 nm and *λ*_*S*_ = 1490 nm. Panels (e) and (f) depict the corresponding normalized lateral and axial profiles, respectively, with dashed lines indicating the half-maximum level; red curves correspond to case 2, and blue curves correspond to case 1. Note that both case 1 and case 2 target a Raman shift near 3000 cm^−1^ in the C–H stretching region. Scale bars, 0.4 *μ*m.

The simulation shows that the effective CARS interaction volume of the conventional configuration [Figs. 7(a) and 7(b)] was tighter than that from the long-Stokes configuration [Figs. 7(c) and 7(d)], indicating that the longer Stokes wavelength broadens the effective CARS PSF. Using the lateral profiles in Fig. 7(e) and the axial profiles in Fig. 7(f), we compared the spatial confinement of the two configurations. These profiles show that both the lateral and axial FWHMs increased by a factor of 1.34× in case 2 relative to case 1, which confirms a reduced spatial resolution for the long-Stokes configuration.

#### 2. Depth-dependent CARS signal generation

The long-Stokes wavelength of the OPG is also expected to reduce depth-dependent CARS signal generation. This reduction arises because water within tissues exhibits substantially higher absorption near 1490 nm than near conventional Stokes wavelengths around 1030–1100 nm.^100^ Consider the absorption coefficient of water, *μ*_*a*_, which quantifies absorption probability per unit path length^101^ and increases from $\approx 0.20\;{cm}^{-1}$ near 1030 nm to $\approx 29.52\;{cm}^{-1}$ near 1490 nm, with cm^−1^ here referring to inverse propagation distance. This corresponds to an ≈148-fold increase in water-mediated attenuation of the Stokes beam. From the Beer–Lambert law and assuming that attenuation arises only from absorption events, the 1490 nm intensity relative to a 1030 nm beam is *I*_*S*1490_(*z*)/*I*_*S*1030_(*z*) = exp[−(*μ*_*a*,1490_ − *μ*_*a*,1030_)*z*]. At depths of *z* = 100, 250, and 500 *μ*m, this ratio yields values of ≈0.75, ≈0.48, and ≈0.23, respectively. Because CARS scales linearly with the Stokes intensity, attenuation of the Stokes beam reduces depth-dependent CARS signal generation. Thus, the long-Stokes beam derived from the OPG will suffer stronger attenuation in hydrated tissues, reducing the CARS imaging depth relative to conventional CARS configurations.

Overall, the longer Stokes wavelength achieved with the OPG compromises spatial resolution and penetration depth. However, these limitations do not preclude biologically relevant imaging. In fact, the simulated CARS PSF (Fig. 7) remains sufficiently confined to support cellular-level contrast, and the experimentally acquired images demonstrate that the OPG-based configuration successfully resolves tissue-specific morphological and chemical features (see Secs. IV C 2, IV C 3, and V). Thus, the long-Stokes configuration represents a trade-off: it sacrifices spatial resolution and imaging depth in favor of reduced electronic-resonances and tissue damage, as well as lower contributions from the nonresonant background.

### C. Vibrational spectroscopy and microscopy based on CARS

Using a narrowband of the fundamental 1 beam as the pump (*ω*_pu_) and the OPG signal as the Stokes (*ω*_*S*_), we acquired CARS spectra from spectroscopic-grade solvents, freshly excised and minimally processed rodent tissues. These measurements confirm that the OPG drives vibrational coherences in the C–H stretching region (2800–3000 cm^−1^).

#### 1. Spectroscopic validation: CARS spectra of benchmark solvents

**FIG. 8.**
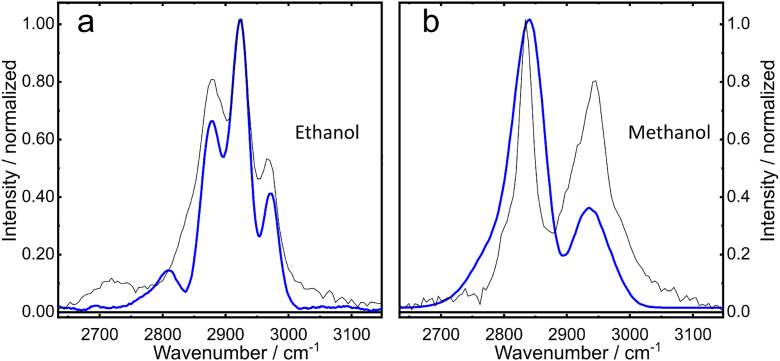
CARS spectra of organic solvents: (a) ethanol and (b) methanol. The black curves are the corresponding spontaneous Raman spectra. The CARS spectra were acquired using the Stokes beam generated in the PPLN using the Λ_1_ grating.

To establish spectroscopic capability, we performed CARS measurements from benchmark spectroscopic-grade solvents: ethanol (C_2_H_5_OH) and methanol (CH_3_OH), small organic molecules that possess abundant C–H bonds with strong Raman activity. Figure 8 shows the results of these experiments. By shifting the pump, Stokes, and anti-Stokes wavelengths far from electronic resonances, the OPG mitigates nonresonant and fluorescence backgrounds. This configuration reduces photodamage and yields CARS spectra with minimal distortion, closely matching tabulated Raman traces. Importantly, we observed strong attenuation of the Stokes beam as it propagated through the solvents. This attenuation may originate from vibrational overtone absorption rather than from electronic transitions, most notably O–H overtones in the 1400–1500 nm band. Nevertheless, these data demonstrate the broadband capability of our source to retrieve molecule-specific CARS spectra, thereby validating the OPG for spectroscopic implementations.

#### 2. Label-free CARS imaging of freshly excised rodent tissues—narrowband detection

**FIG. 9.**
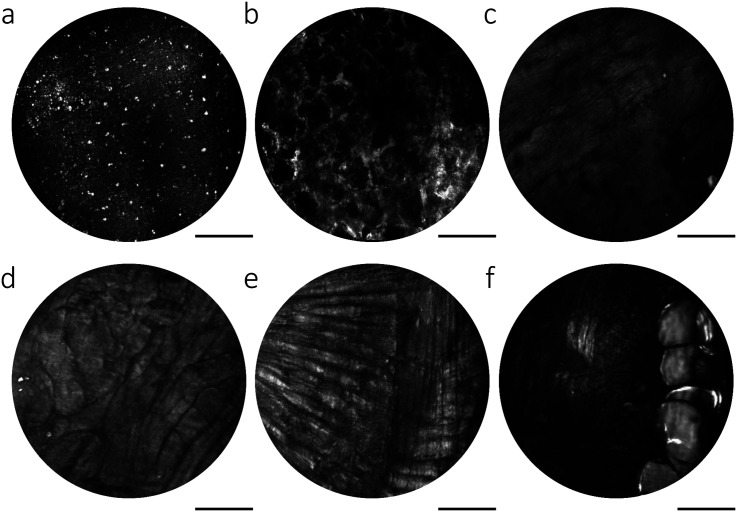
Narrowband CARS images of freshly excised mouse organs. The images integrate CARS radiation over 2800–3000 cm^−1^. (a) liver, (b) lungs, (c) heart, (d) kidney, (e) tongue, and (f) adipose tissue. The organs were imaged intact (i.e., without post-harvest sectioning or embedding). Scale bars: 30 *μ*m. Pixel dwell time: 20 *μ*s. The circular appearance is a visualization choice.

To demonstrate that the OPG can elicit vibrational coherences in pristine biological specimens, we imaged several freshly excised murine tissues (Fig. 9), including the liver, lungs, heart, kidney, tongue, and peri-muscular fat, targeting molecular vibrations in the C–H range. In these experiments, the CARS signal was epi-detected and integrated over this band using a single PMT. Although we sacrificed spectral resolution in favor of speed, the CARS contrast reveals the microscopic landscape within the specimens, exposing morphofunctional features at high speed. The images in Fig. 9 establish the capacity of the OPG to provide vibrational contrast for rapid imaging of intact specimens, enabling high-throughput morphofunctional screening of the tissue microenvironment without exogenous labels.

#### 3. Label-free histology through broadband CARS microscopy

**FIG. 10.**
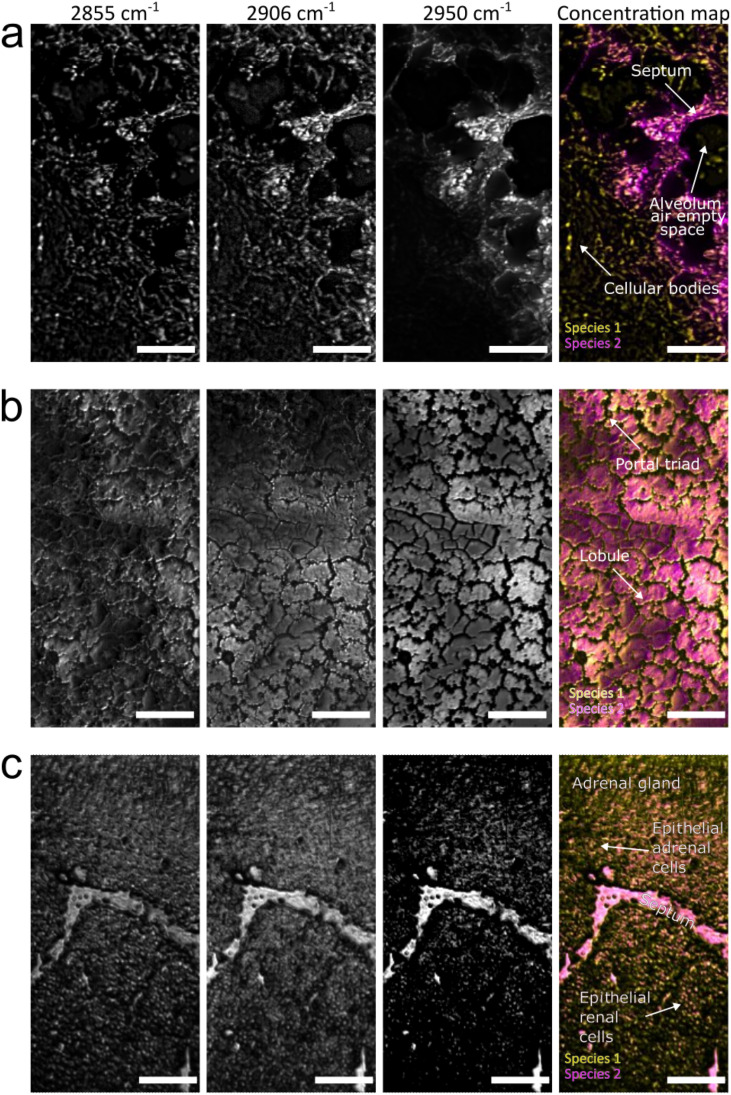
Digital histology based on broadband CARS microscopy of fresh-frozen murine (a) lungs, (b) liver, and (c) kidney tissues. The composite images on the right show the concentration maps extracted from CARS hypercubes derived from datasets with 300 × 150 pixels × 560 spectral points, each acquired with the spectrometer operating at a time constant of 1 ms (the lowest integration time available). The scale bars in (a) and (c) correspond to 40 *μ*m, while those in (b) correspond to 380 *μ*m.

Next, we validated the OPG performance for label-free, non-destructive imaging based on broadband CARS by acquiring hyperspectral datasets of fresh-frozen murine tissue. By raster-scanning the specimens and recording spectral traces at each position, the system generated a hyperspectral cube, i.e., a three-dimensional dataset containing the spatial distribution of the CARS signal at different wavelengths (or corresponding wavenumbers, cm^−1^). These data enabled image formation at selected vibrational modes [Figs. 10(a)–10(c)] and facilitated chemometric analysis, an algorithm that extracted relative concentration maps (fourth column in Fig. 10) and identified two dominant species (characteristic spectra in Fig. 3 of the supplementary material).

Although the features observed in Fig. 10 correspond to compartments visible in matched histological references (Fig. 4 of the supplementary material), the CARS images reveal structural and chemical detail without the need for exogenous labeling. These results position the OPG as a viable source for broadband CARS microscopy and underscore its potential for label-free digital histology.

## V. LABEL-FREE MULTIMODAL NONLINEAR MICROSCOPY

Biological specimens are highly heterogeneous. Therefore, a single contrast may not suffice to elucidate their structural and functional features. In this context, a multimodal approach is advantageous because it enables spatial separation of constituents. In addition, multimodal imaging enables quantification of how different light–matter interactions—associated with specific biological processes—are correlated across an image. Realizing such multimodal measurements introduces two technical challenges: one pertains to the detection chain, and the other pertains to the laser source. Here, we demonstrate that the OPG offers a simple solution to drive multimodal label-free nonlinear microscopy.

**FIG. 11.**
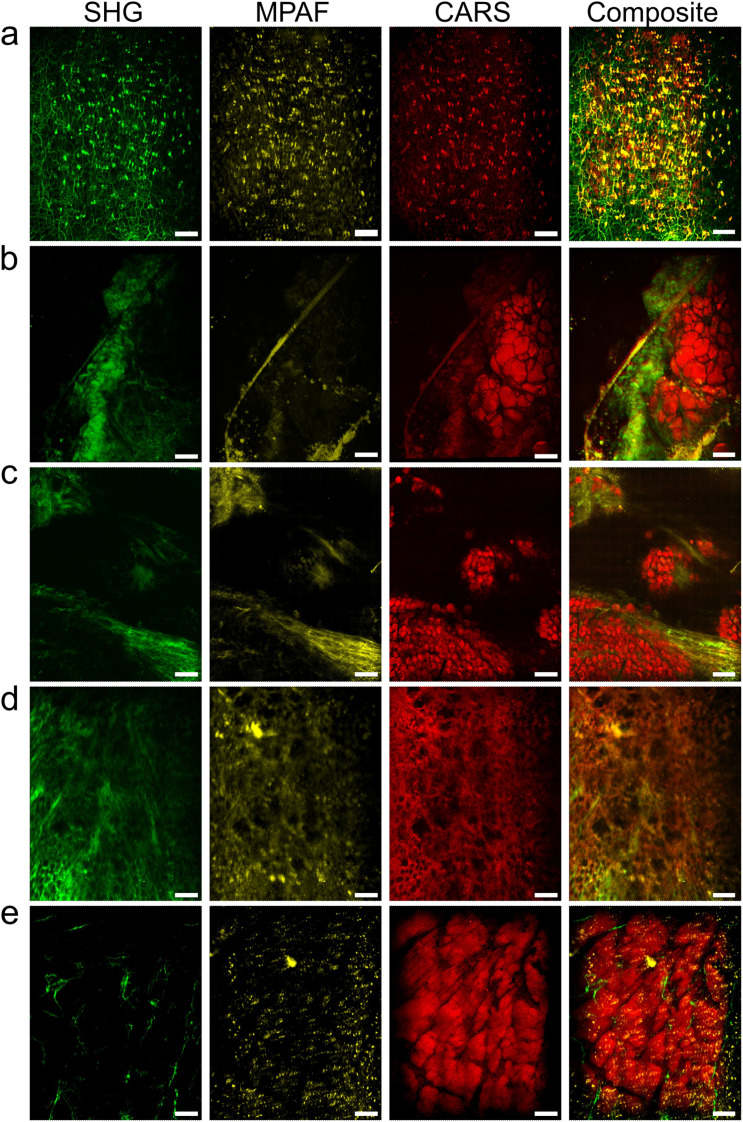
Multimodal label-free imaging of freshly excised rodent tissues: (a) liver, (b) abdominal adipose tissue, (c) mammary gland, (d) lung, and (e) heart. All tissues were collected from a mouse, except the mammary gland tissue shown in (c), which was collected from a rat. Scale bars: 40 *μ*m in (a), (b), (d), 150 *μ*m in (c), and 50 *μ*m in (e). Pixel dwell time: 20 *μ*s. Color coding: SHG, green; MPAF, yellow; CARS, red.

As shown in Sec. IV C, the OPG generates vibrational contrast via CARS, which requires a 1030 nm pump and a Stokes field near 1500 nm. By exploiting the capabilities of the pump to efficiently drive two-photon processes, we collect SHG and MPAF in parallel with CARS. Although this approach sacrifices spectral resolution, it provides complementary, co-registered contrasts, thereby enabling multimodal imaging. Using this strategy, we imaged a variety of freshly excised rodent tissues (Fig. 11), co-registering SHG, MPAF, and CARS signals.

This approach relies on the SHG channel to probe noncentrosymmetric structures, potentially revealing microscopic features related to collagen, such as extracellular matrix components and collagen-rich fibrous septa. The MPAF channel registers endogenous fluorophores, likely elastin and flavoproteins, and, in the case of the liver specimen [Fig. 11(a)], lipopigments. The CARS channel effectively reveals compartments containing C–H bonds, notably lipid-rich structures, such as adipocytes in adipose tissue [Fig. 11(b)] and in the mammary gland [Fig. 11(c)], as well as lipid droplets in the liver [Fig. 11(a)] and surfactant in the lungs [Fig. 11(d)]. In addition, owing to the relatively broad bandwidth of the Stokes beam, the CARS channel may also reveal protein-rich features, as exemplified in heart tissue [Fig. 11(e)].

The composite multimodal images presented in Fig. 11 provide richer morphofunctional information than any individual channel alone. Most importantly, they establish the OPG as a viable laser source for label-free multimodal nonlinear optical microscopy.

## VI. DISCUSSION AND CONCLUSION

In this article, we introduced an optical source for nonlinear optical microscopy and spectroscopy based on an OPG. We provided a description of its architecture and performance, including its spectral, temporal, and spatial properties, and its noise characteristics. We validated its spectroscopic capability by acquiring CARS spectra from benchmark solvents, attaining results that closely match tabulated Raman spectra in the C–H band. We demonstrated tissue imaging for histological analysis using both broadband and narrowband CARS contrasts. Equally important, we demonstrated label-free multimodal nonlinear microscopy by co-registering SHG, MPAF, and CARS. Collectively, these results position the OPG as a powerful and versatile source for nonlinear optical microspectroscopy, offering a compact, simple, and cost-effective design.

Its simplicity and tunability make the OPG a highly adaptable setup, as these features allow the nonlinear microscope to match the spectroscopic properties of the specimen. Our implementation strategically shifts the pump, Stokes, and CARS wavelengths far from electronic resonances to mitigate both the nonresonant background and photodamage. Such adaptability also allows spectral shifting the CARS signal away from strong fluorescence backgrounds, a relevant consideration for both label-based and plant imaging^102^—chlorophyll emission, which can overwhelm CARS detection in the visible range (see Fig. 5 of the supplementary material). While in this work, we prioritized readily accessible signals, future implementations could capitalize on the ability of the OPG to generate additional wavelengths, fields that, alone or in tandem, could enable a broader set of nonlinear contrasts.

Naturally, like all sources summarized in Table I, the OPG has limitations. Chief among these are limited tolerance to high pump power, noise degradation, and relatively long emission wavelength. The first limitation constrains pump power in the crystal to avoid damage to the poled gratings, the second can limit optical heterodyne detection (e.g., pump–probe schemes), and the third may reduce the achievable imaging resolution and penetration depth. Nevertheless, the available signal power was sufficient to drive our microscope, homodyne-based detection supported high-speed imaging, and the attainable resolution still revealed cellular-level detail.

To conclude, we presented an OPG-based excitation source optimized for label-free multimodal nonlinear microscopy. We detailed its optical properties and performance and demonstrated its application to nonlinear microspectroscopy. This approach offers a compact, cost-effective design that facilitates translation and broader adoption of label-free multimodal nonlinear microscopy.

## SUPPLEMENTARY MATERIAL

See the supplementary material for descriptions of animals, preparation of frozen tissue sections by cryotomy for imaging, histological staining, spontaneous Raman spectroscopy, spectral traces of the dominant constituents in Fig. 10, matched histological sections for the broadband CARS experiments, and the benefits of spectrally shifting the excitation in multimodal nonlinear microscopy.

## Data Availability

The data that support the findings of this study are available from the corresponding author upon reasonable request.

## References

[1] S. Mukamel, Principles of Nonlinear Optical Spectroscopy, 1st ed. (Oxford University Press, 1995), p. 564.

[2] Y. R. Shen, The Principles of Nonlinear Optics, 1st ed. (John Wiley and Sons, 2002), p. 576.

[3] E. O. Potma, Foundations of Nonlinear Optical Microscopy (John Wiley and Sons, 2024).

[4] A. De la Cadena, J. Park, J. Shi, and S. A. Boppart, “On the importance of simultaneous label-free multimodal nonlinear optical imaging for biomedical applications,” APL Photonics 10, 110902 (2025). 10.1063/5.0289864 41282567 PMC12632184

[5] W. R. Zipfel, R. M. Williams, and W. W. Webb, “Nonlinear magic: Multiphoton microscopy in the biosciences,” Nat. Biotechnol. 21, 1369–1377 (2003). 10.1038/nbt899 14595365

[6] W. R. Zipfel, R. M. Williams, R. Christie, A. Y. Nikitin, B. T. Hyman, and W. W. Webb, “Live tissue intrinsic emission microscopy using multiphoton-excited native fluorescence and second harmonic generation,” Tech. Rep. 100, 7075–7080 (2003). 10.1073/pnas.0832308100 PMC165832 12756303

[7] P. Campagnola, “Second harmonic generation imaging microscopy: Applications to diseases diagnostics,” Anal. Chem. 83, 3224–3231 (2011). 10.1021/ac1032325 21446646 PMC3104727

[8] M. Ji, D. A. Orringer, C. W. Freudiger, S. Ramkissoon, X. Liu, D. Lau, A. J. Golby, I. Norton, M. Hayashi, N. Y. R. Agar, G. S. Young, C. Spino, S. Santagata, S. Camelo-Piragua, K. L. Ligon, O. Sagher, and X. S. Xie, “Rapid, label-free detection of brain tumors with stimulated Raman scattering microscopy,” Sci. Transl. Med. 5, 201ra119 (2013). 10.1126/scitranslmed.3005954 PMC3806096 24005159

[9] S. You, Y. Sun, E. J. Chaney, Y. Zhao, J. Chen, S. A. Boppart, and H. Tu, “Slide-free virtual histochemistry (Part I): Development via nonlinear optics,” Biomed. Opt. Express 9, 5240 (2018). 10.1364/boe.9.005240 30460125 PMC6238939

[10] B. Talone, A. Bresci, F. Manetti, F. Vernuccio, A. De la Cadena, C. Ceconello, M. L. Schiavone, S. Mantero, C. Menale, R. Vanna, G. Cerullo, C. Sobacchi, and D. Polli, “Label-free multimodal nonlinear optical microscopy reveals features of bone composition in pathophysiological conditions,” Front. Bioeng. Biotechnol. 10, 1042680 (2022). 10.3389/fbioe.2022.1042680 36483771 PMC9723390

[11] C. Xu, M. Nedergaard, D. J. Fowell, P. Friedl, and N. Ji, “Multiphoton fluorescence microscopy for in vivo imaging,” Cell 187, 4458–4487 (2024). 10.1016/j.cell.2024.07.036 39178829 PMC11373887

[12] M. Nuriya, “Uses of coherent Raman scattering microscopy in neuroscience,” Front. Neurosci. 19, 1715954 (2026). 10.3389/fnins.2025.1715954 41694718 PMC12894379

[13] R. W. Boyd, in Nonlinear Optics, 3rd ed., edited by R. W. Boyd (Academic Press, 2003).

[14] M. D. Duncan, J. Reintjes, and T. J. Manuccia, “Scanning coherent anti-Stokes Raman microscope,” Opt. Lett. 7, 350 (1982). 10.1364/ol.7.000350 19714017

[15] W. Denk, J. H. Strickler, and W. W. Webb, “Two-photon laser scanning fluorescence microscopy,” Science 248, 73–76 (1990). 10.1126/science.2321027 2321027

[16] P. F. Moulton, “Spectroscopic and laser characteristics of *Ti*: *Al*_2_*O*_3_,” J. Opt. Soc. Am. B 3, 125 (1986). 10.1364/josab.3.000125

[17] P. Hammerling, A. B. Budgor, and A. Pinto, “Tunable solid state lasers,” in *Proceedings of the First International Conference La Jolla, California, June 13–15, 1984* (Springer, 2013), Vol. 47.

[18] W. G. Fisher, E. A. Wachter, M. Armas, and C. Seaton, “Titanium: Sapphire laser as an excitation source in two-photon spectroscopy,” Appl. Spectrosc. 51, 218–226 (1997). 10.1366/0003702971939910

[19] C. Y. Dong, P. T. So, T. French, and E. Gratton, “Fluorescence lifetime imaging by asynchronous pump-probe microscopy,” Biophys. J. 69, 2234–2242 (1995). 10.1016/s0006-3495(95)80148-7 8599631 PMC1236462

[20] N. P. Barnes, “Diode-pumped solid-state lasers,” J. Appl. Phys. 44, 230–237 (1973). 10.1063/1.1661867

[21] R. L. Byer, “Diode laser—Pumped solid-state lasers,” Science 239, 742–747 (1988). 10.1126/science.239.4841.742 17832940

[22] A. Zumbusch, G. R. Holtom, and X. S. Xie, “Three-dimensional vibrational imaging by coherent anti-Stokes Raman scattering,” Phys. Rev. Lett. 82, 4142–4145 (1999). 10.1103/physrevlett.82.4142

[23] J.-X. Cheng, A. Volkmer, L. D. Book, and X. S. Xie, “An epi-detected coherent anti-Stokes Raman scattering (E-CARS) microscope with high spectral resolution and high sensitivity,” J. Phys. Chem. B 105, 1277–1280 (2001). 10.1021/jp003774a

[24] M. Marangoni, A. Gambetta, C. Manzoni, V. Kumar, R. Ramponi, and G. Cerullo, “Fiber-format CARS spectroscopy by spectral compression of femtosecond pulses from a single laser oscillator,” Opt. Lett. 34, 3262 (2009). 10.1364/ol.34.003262 19881561

[25] A. F. Pegoraro, A. Ridsdale, D. J. Moffatt, J. P. Pezacki, B. K. Thomas, L. Fu, L. Dong, M. E. Fermann, and A. Stolow, “All-fiber CARS microscopy of live cells,” Opt. Express 17, 20700 (2009). 10.1364/oe.17.020700 19997300

[26] C. Xu and F. W. Wise, “Recent advances in fibre lasers for nonlinear microscopy,” Nat. Photonics 7, 875–882 (2013). 10.1038/nphoton.2013.284 PMC3887125 24416074

[27] M. Brinkmann, A. Fast, T. Hellwig, I. Pence, C. L. Evans, and C. Fallnich, “Portable all-fiber dual-output widely tunable light source for coherent Raman imaging,” Biomed. Opt. Express 10, 4437 (2019). 10.1364/boe.10.004437 31565500 PMC6757451

[28] V. Mazeika, K. Mirsanaye, L. U. Castaño, S. Krouglov, M. Alizadeh, M. Maciulis, L. Kontenis, V. Karabanovas, and V. Barzda, “Double Stokes polarimetric microscopy for chiral fibrillar aggregates,” Sci. Rep. 15, 4464 (2025). 10.1038/s41598-025-86893-0 39915558 PMC11803116

[29] A. De la Cadena, C. A. Renteria, E. Aksamitiene, and S. A. Boppart, “Label-free hyperspectral multiphoton microscopy,” Opt. Lett. 50, 1484 (2025). 10.1364/ol.547032 40019961 PMC12118598

[30] C. Hönninger, R. Paschotta, M. Graf, F. Morier-Genoud, G. Zhang, M. Moser, S. Biswal, J. Nees, A. Braun, G. A. Mourou, I. Johannsen, A. Giesen, W. Seeber, and U. Keller, “Ultrafast ytterbium-doped bulk lasers and laser amplifiers,” Appl. Phys. B: Lasers Opt. 69, 3–17 (1999). 10.1007/s003400050762

[31] A. Lucca, M. Jacquemet, F. Druon, F. Balembois, P. Georges, P. Camy, J. L. Doualan, and R. Moncorgé, “High-power tunable diode-pumped Yb_3+_:CaF_2_ laser,” Opt. Lett. 29, 1879 (2004). 10.1364/ol.29.001879 15357346

[32] F. Ganikhanov, S. Carrasco, X. Sunney Xie, M. Katz, W. Seitz, and D. Kopf, “Broadly tunable dual-wavelength light source for coherent anti-Stokes Raman scattering microscopy,” Opt. Lett. 31, 1292 (2006). 10.1364/ol.31.001292 16642089

[33] N. Coluccelli, D. Viola, V. Kumar, A. Perri, M. Marangoni, G. Cerullo, and D. Polli, “Tunable 30 fs light pulses at 1 w power level from a Yb-pumped optical parametric oscillator,” Opt. Lett. 42, 4545 (2017). 10.1364/ol.42.004545 29088209

[34] A. De la Cadena, C. M. Valensise, M. Marangoni, G. Cerullo, and D. Polli, “Broadband stimulated Raman scattering microscopy with wavelength-scanning detection,” J. Raman Spectrosc. 51, 1951–1959 (2020). 10.1002/jrs.5816 33132486 PMC7586786

[35] H. Tu and S. A. Boppart, “Coherent fiber supercontinuum for biophotonics,” Laser Photonics Rev. 7, 628–645 (2013). 10.1002/lpor.201200014 PMC3864867 24358056

[36] K. Wang, N. G. Horton, K. Charan, and C. Xu, “Advanced fiber soliton sources for nonlinear deep tissue imaging in biophotonics,” IEEE J. Sel. Top. Quantum Electron. 20, 6800311 (2014). 10.1109/JSTQE.2013.2276860

[37] C. H. Camp Jr, Y. J. Lee, J. M. Heddleston, C. M. Hartshorn, A. R. H. Walker, J. N. Rich, J. D. Lathia, and M. T. Cicerone, “High-speed coherent Raman fingerprint imaging of biological tissues,” Nat. Photonics 8, 627–634 (2014). 10.1038/nphoton.2014.145 25621002 PMC4304702

[38] H. Tu, Y. Liu, D. Turchinovich, M. Marjanovic, J. K. Lyngsø, J. Lægsgaard, E. J. Chaney, Y. Zhao, S. You, W. L. Wilson, B. Xu, M. Dantus, and S. A. Boppart, “Stain-free histopathology by programmable supercontinuum pulses,” Nat. Photonics 10, 534–540 (2016). 10.1038/nphoton.2016.94 27668009 PMC5031149

[39] S. You, H. Tu, E. J. Chaney, Y. Sun, Y. Zhao, A. J. Bower, Y.-Z. Liu, M. Marjanovic, S. Sinha, Y. Pu, and S. A. Boppart, “Intravital imaging by simultaneous label-free autofluorescence-multiharmonic microscopy,” Nat. Commun. 9, 2125 (2018). 10.1038/s41467-018-04470-8 29844371 PMC5974075

[40] K. K. D. Tan, A. De la Cadena, E. Aksamitiene, A. Ho, and S. A. Boppart, “Simultaneous label-free autofluorescence multi-harmonic microscopy,” J. Visualized Exp. 222, 3791 (2025). 10.3791/68637 PMC12624867 40952928

[41] K. Wang, D. Kobat, N. Horton, and C. Xu, “High-energy soliton pulse generation in a photonic crystal rod and its application to three-photon microscopy,” in *Conference on Lasers and Electro-Optics* (OSA, 2012), pp. CM2J–3.

[42] D. Polli, G. Grancini, J. Clark, M. Celebrano, T. Virgili, G. Cerullo, and G. Lanzani, “Nanoscale imaging of the interface dynamics in polymer blends by femtosecond pump-probe confocal microscopy,” Adv. Mater. 22, 3048–3051 (2010). 10.1002/adma.200904387 20578123

[43] F. Vernuccio, A. Bresci, B. Talone, A. De la Cadena, C. Ceconello, S. Mantero, C. Sobacchi, R. Vanna, G. Cerullo, and D. Polli, “Fingerprint multiplex CARS at high speed based on supercontinuum generation in bulk media and deep learning spectral denoising,” Opt. Express 30, 30135 (2022). 10.1364/oe.463032 36242123

[44] C. Ceconello, F. Vernuccio, A. De la Cadena, A. Bresci, F. Manetti, S. Das, R. Vanna, G. Cerullo, and D. Polli, “Wide-field broadband cars microscopy,” EPJ Web Conf. 266, 08001 (2022). 10.1051/epjconf/202226608001

[45] F. Vernuccio, R. Vanna, C. Ceconello, A. Bresci, F. Manetti, S. Sorrentino, S. Ghislanzoni, F. Lambertucci, O. Motiño, I. Martins, G. Kroemer, I. Bongarzone, G. Cerullo, and D. Polli, “Full-spectrum CARS microscopy of cells and tissues with ultrashort white-light continuum pulses,” J. Phys. Chem. B 127, 4733 (2023). 10.1021/acs.jpcb.3c01443 37195090 PMC10240501

[46] A. De la Cadena, J. Park, K. F. Tehrani, C. A. Renteria, G. L. Monroy, and S. A. Boppart, “Simultaneous label-free autofluorescence multi-harmonic microscopy driven by the supercontinuum generated from a bulk nonlinear crystal,” Biomed. Opt. Express 15, 491 (2024). 10.1364/boe.504832 38404303 PMC10890845

[47] T. Steinle, V. Kumar, M. Floess, A. Steinmann, M. Marangoni, C. Koch, C. Wege, G. Cerullo, and H. Giessen, “Synchronization-free all-solid-state laser system for stimulated Raman scattering microscopy,” Light: Sci. Appl. 5, e16149 (2016). 10.1038/lsa.2016.149 30167121 PMC6059832

[48] Z. Heiner, V. Petrov, and M. Mero, “Compact, high-repetition-rate source for broadband sum-frequency generation spectroscopy,” APL Photonics 2, 066102 (2017). 10.1063/1.4983691

[49] K. Guesmi, L. Abdeladim, S. Tozer, P. Mahou, T. Kumamoto, K. Jurkus, P. Rigaud, K. Loulier, N. Dray, P. Georges, M. Hanna, J. Livet, W. Supatto, E. Beaurepaire, and F. Druon, “Dual-color deep-tissue three-photon microscopy with a multiband infrared laser,” Light: Sci. Appl. 7, 12 (2018). 10.1038/s41377-018-0012-2 30839589 PMC6107000

[50] Y. Sun, H. Tu, and S. A. Boppart, “Nonlinear optical imaging by detection with optical parametric amplification (invited paper),” J. Innovative Opt. Health Sci. 16, 2245001 (2023). 10.1142/S1793545822450018 PMC10426456 37583790

[51] F. Vernuccio, A. Benachir, E. M. Fantuzzi, B. Morel, S. Bux, E. Martin, J. Villanueva, Y. Pertot, N. Thiré, S. Heuke, and H. Rigneault, “Dual picosecond fast tunable optical parametric amplifier laser system for wide-field nonlinear optical microscopy,” APL Photonics 9, 096113 (2024). 10.1063/5.0221283

[52] G. Murray, J. Field, M. Xiu, Y. Farah, L. Wang, O. Pinaud, and R. Bartels, “Aberration free synthetic aperture second harmonic generation holography,” Opt. Express 31, 32434 (2023). 10.1364/oe.496083 37859047

[53] D. Tokarz, R. Cisek, M. N. Wein, R. Turcotte, C. Haase, S.-C. A. Yeh, S. Bharadwaj, A. P. Raphael, H. Paudel, C. Alt, T.-M. Liu, H. M. Kronenberg, and C. P. Lin, “Intravital imaging of osteocytes in mouse calvaria using third harmonic generation microscopy,” PLoS One 12, e0186846 (2017). 10.1371/journal.pone.0186846 29065178 PMC5655444

[54] Y. Farah, G. Murray, J. Field, M. Varughese, L. Wang, O. Pinaud, and R. Bartels, “Synthetic spatial aperture holographic third harmonic generation microscopy,” Optica 11, 693 (2024). 10.1364/optica.521088

[55] I. Georgakoudi and K. P. Quinn, “Optical imaging using endogenous contrast to assess metabolic state,” Annu. Rev. Biomed. Eng. 14, 351–367 (2012). 10.1146/annurev-bioeng-071811-150108 22607264

[56] C. A. Renteria, J. Park, C. Zhang, J. E. Sorrells, R. R. Iyer, K. F. Tehrani, A. De la Cadena, and S. A. Boppart, “Large field-of-view metabolic profiling of murine brain tissue following morphine incubation using label-free multiphoton microscopy,” J. Neurosci. Methods 408, 110171 (2024). 10.1016/j.jneumeth.2024.110171 38777156 PMC12047187

[57] T. Meyer, M. Schmitt, B. Dietzek, and J. Popp, “Accumulating advantages, reducing limitations: Multimodal nonlinear imaging in biomedical sciences—The synergy of multiple contrast mechanisms,” J. Biophot. 6, 887–904 (2013). 10.1002/jbio.201300176 24259267

[58] S. Heuke and H. Rigneault, “Coherent Stokes Raman scattering microscopy (CSRS),” Nat. Commun. 14, 3337 (2023). 10.1038/s41467-023-38941-4 37286641 PMC10247746

[59] S. P. Laptenok, V. P. Rajamanickam, L. Genchi, T. Monfort, Y. Lee, I. I. Patel, A. Bertoncini, and C. Liberale, “Fingerprint-to-CH stretch continuously tunable high spectral resolution stimulated Raman scattering microscope,” J. Biophot. 12, e201900028 (2019). 10.1002/jbio.201900028 31081280

[60] K. Bae, W. Zheng, K. Lin, S. W. Lim, Y. K. Chong, C. Tang, N. K. King, C. B. Ti Ang, and Z. Huang, “Epi-detected hyperspectral stimulated Raman scattering microscopy for label-free molecular subtyping of glioblastomas,” Anal. Chem. 90, 10249–10255 (2018). 10.1021/acs.analchem.8b01677 30070837

[61] K. Bae, L. Xin, W. Zheng, C. Tang, B.-T. Ang, and Z. Huang, “Mapping the intratumoral heterogeneity in glioblastomas with hyperspectral stimulated Raman scattering microscopy,” Anal. Chem. 93, 2377–2384 (2021). 10.1021/acs.analchem.0c04262 33443405

[62] A. De la Cadena, F. Vernuccio, A. Ragni, G. Sciortino, R. Vanna, C. Ferrante, N. Pediconi, C. Valensise, L. Genchi, S. P. Laptenok, A. Doni, M. Erreni, T. Scopigno, C. Liberale, G. Ferrari, M. Sampietro, G. Cerullo, and D. Polli, “Broadband stimulated Raman imaging based on multi-channel lock-in detection for spectral histopathology,” APL Photonics 7, 076104 (2022). 10.1063/5.0093946

[63] D. Davydova, A. De la Cadena, D. Akimov, and B. Dietzek, “Transient absorption microscopy: Advances in chemical imaging of photoinduced dynamics,” Laser Photonics Rev. 10, 62–81 (2016). 10.1002/lpor.201500181

[64] D. Davydova, A. De la Cadena, S. Demmler, J. Rothhardt, J. Limpert, T. Pascher, D. Akimov, and B. Dietzek, “Ultrafast transient absorption microscopy: Study of excited state dynamics in PtOEP crystals,” Chem. Phys. 464, 69–77 (2016). 10.1016/j.chemphys.2015.11.006

[65] A. De la Cadena, D. Davydova, T. Tolstik, C. Reichardt, S. Shukla, D. Akimov, R. Heintzmann, J. Popp, and B. Dietzek, “Ultrafast in cellulo photoinduced dynamics processes of the paradigm molecular light switch [ru(bpy)2dppz]2+,” Sci. Rep. 6, 33547 (2016). 10.1038/srep33547 27644587 PMC5028833

[66] A. Hanninen, M. W. Shu, and E. O. Potma, “Hyperspectral imaging with laser-scanning sum-frequency generation microscopy,” Biomed. Opt. Express 8, 4230 (2017). 10.1364/boe.8.004230 28966861 PMC5611937

[67] J. H. Magnus, L. T. Nguyen, E. G. Alevy, T. W. Sawyer, S. D. Crossley, and K. Kieu, “Fifth-harmonic and five-photon excitation fluorescence multiphoton microscopy,” Opt. Lett. 50, 3931 (2025). 10.1364/ol.566910 40512910

[68] S. Lambert-Girard, M. Allard, M. Piché, and F. Babin, “Broadband and tunable optical parametric generator for remote detection of gas molecules in the short and mid-infrared,” Appl. Opt. 54, 2594 (2015). 10.1364/ao.54.002594 25967164

[69] S. C. Kumar, B. Nandy, and M. Ebrahim-Zadeh, “Performance studies of high-average-power picosecond optical parametric generation and amplification in MgO:PPLN at 80 MHz,” Opt. Express 28, 39189 (2020). 10.1364/oe.411276 33379474

[70] H. Qiao, K. Zhong, F. Li, X. Zhang, S. Wang, Y. Zheng, D. Xu, Q. Sheng, W. Shi, and J. Yao, “Efficient MW-peak-power kHz-repetition-rate sub-nanosecond optical parametric generator tunable from near- to mid-infrared,” Opt Laser. Technol. 151, 108010 (2022). 10.1016/j.optlastec.2022.108010

[71] J. Banys, S. Armalytė, J. Pimpė, O. Balachninaitė, V. Jarutis, and J. Vengelis, “Subnanosecond microlaser pumped fan-out grating design MgO: PPLN optical parametric generator continuously tunable from near-to mid-infrared,” Opt Laser. Technol. 171, 110433 (2024). 10.1016/j.optlastec.2023.110433

[72] J. W. Haus and P. E. Powers, Fundamentals of Nonlinear Optics (CRC Press, 2017).

[73] M. M. Fejer, G. A. Magel, D. H. Jundt, and R. L. Byer, “Quasi-phase-matched second harmonic generation: Tuning and tolerances,” IEEE J. Quantum Electron. 28, 2631–2654 (1992). 10.1109/3.161322

[74] D. S. Hum and M. M. Fejer, “Quasi-phasematching,” C. R. Phys. 8, 180–198 (2006). 10.1016/j.crhy.2006.10.022

[75] C. Manzoni and G. Cerullo, “Design criteria for ultrafast optical parametric amplifiers,” J. Opt. 18, 103501 (2016). 10.1088/2040-8978/18/10/103501

[76] A. De la Cadena, E. Aksamitiene, and S. A. Boppart, “Unified vibrational and multiphoton label-free nonlinear microscopy for simultaneous chemical and structural imaging,” IEEE J. Sel. Top. Quantum Electron. 32, 1–12 (2026). 10.1109/jstqe.2025.3650148 PMC12971071 41809093

[77] C. H. Camp Jr and M. T. Cicerone, “Chemically sensitive bioimaging with coherent Raman scattering,” Nat. Photonics 9, 295–305 (2015). 10.1038/nphoton.2015.60

[78] S. N. Arkhipov, I. Saytashev, and M. Dantus, “Intravital imaging study on photodamage produced by femtosecond near-infrared laser pulses *in vivo*,” Photochem. Photobiol. 92, 308–313 (2016). 10.1111/php.12572 26814684 PMC4963309

[79] D. J. Kane and R. Trebino, “Characterization of arbitrary femtosecond pulses using frequency-resolved optical gating,” IEEE J. Quantum Electron. 29, 571–579 (1993). 10.1109/3.199311

[80] R. Trebino, Frequency-Resolved Optical Gating: The Measurement of Ultrashort Laser Pulses (Springer US, 2000).

[81] O. Svelto, Principles of Lasers (Springer US, 2010).

[82] D. Polli, V. Kumar, C. M. Valensise, M. Marangoni, and G. Cerullo, “Broadband coherent Raman scattering microscopy,” Laser Photonics Rev. 12, 1800020 (2018). 10.1002/lpor.201800020

[83] H. Rigneault and P. Berto, “Tutorial: Coherent Raman light matter interaction processes,” APL Photonics 3, 091101 (2018). 10.1063/1.5030335

[84] R. Vanna, A. De la Cadena, B. Talone, C. Manzoni, M. Marangoni, D. Polli, and G. Cerullo, “Vibrational imaging for label-free cancer diagnosis and classification,” Riv. Nuovo Cimento 45, 107 (2021). 10.1007/s40766-021-00027-6

[85] X. Audier, S. Heuke, P. Volz, I. Rimke, and H. Rigneault, “Noise in stimulated Raman scattering measurement: From basics to practice,” APL Photonics 5, 011101 (2020). 10.1063/1.5129212

[86] A. De la Cadena, F. Vernuccio, B. Talone, A. Bresci, C. Ceconello, S. Das, R. Vanna, G. Cerullo, and D. Polli, “Multiplex chemical imaging based on broadband stimulated Raman scattering microscopy,” J. Visualized Exp. 2022. 10.3791/63709 35938835

[87] Y. Wang, J. Fonseca-Campos, W.-g. Liang, C.-Q. Xu, and I. Vargas-Baca, “Noise analysis of second-harmonic generation in undoped and MgO-doped periodically poled lithium niobate,” Adv. OptoElectron. 2008, 428971. 10.1155/2008/428971

[88] F. C. Cruz, D. L. Maser, T. Johnson, G. Ycas, A. Klose, F. R. Giorgetta, I. Coddington, and S. A. Diddams, “Mid-infrared optical frequency combs based on difference frequency generation for molecular spectroscopy,” Opt. Express 23, 26814 (2015). 10.1364/oe.23.026814 26480192

[89] W. Chen, J. Fan, A. Ge, H. Song, Y. Song, B. Liu, L. Chai, C. Wang, and M. Hu, “Intensity and temporal noise characteristics in femtosecond optical parametric amplifiers,” Opt. Express 25, 31263 (2017). 10.1364/oe.25.031263 29245803

[90] A. Barh, P. J. Rodrigo, L. Meng, C. Pedersen, and P. Tidemand-Lichtenberg, “Parametric upconversion imaging and its applications,” Adv. Opt. Photonics 11, 952 (2019). 10.1364/aop.11.000952

[91] R. Paschotta, “Relative Intensity Noise (RP Photonics Encyclopedia, 2007), see https://www.rp-photonics.com/relative_intensity_noise.html.

[92] H. Rigneault and Y. Ozeki, “Chapter 2—Sensitivity and noise in SRS microscopy,” in Stimulated Raman Scattering Microscopy, edited by J.-X. Cheng, W. Min, Y. Ozeki and D. Polli (Elsevier, 2022), pp. 21–40.

[93] Stimulated Raman Scattering Microscopy: Techniques and Applications, edited by J.-X. Cheng, W. Min, Y. Ozeki and D. Polli, 1st ed. (Elsevier, 2021).

[94] H. Jang, Y. Li, A. A. Fung, P. Bagheri, K. Hoang, D. Skowronska-Krawczyk, X. Chen, J. Y. Wu, B. Bintu, and L. Shi, “Super-resolution SRS microscopy with A-PoD,” Nat. Methods 20, 448–458 (2023). 10.1038/s41592-023-01779-1 36797410 PMC10246886

[95] H. Lin, S. Seitz, Y. Tan, J.-B. Lugagne, L. Wang, G. Ding, H. He, T. J. Rauwolf, M. J. Dunlop, J. H. Connor *et al.*, “Label-free nanoscopy of cell metabolism by ultrasensitive reweighted visible stimulated Raman scattering,” Nat. Methods 22, 1040–1050 (2025). 10.1038/s41592-024-02575-1 39820753 PMC12074879

[96] V. Kumar, A. De la Cadena, A. Perri, F. Preda, N. Coluccelli, G. Cerullo, and D. Polli, “Invited article: Complex vibrational susceptibility by interferometric Fourier transform stimulated Raman scattering,” APL Photonics 3, 092403 (2018). 10.1063/1.5034114

[97] C. M. Valensise, V. Kumar, A. De la Cadena, S. De Silvestri, G. Cerullo, and D. Polli, “Vibrational phase imaging by stimulated Raman scattering via polarization-division interferometry,” Opt. Express 27, 19407–19417 (2019). 10.1364/oe.27.019407 31503700

[98] B. Richards and E. Wolf, “Electromagnetic diffraction in optical systems, II. Structure of the image field in an aplanatic system,” Proc. R. Soc. London Ser. A. Math. Phys. Sci. 253, 358–379 (1959). 10.1098/rspa.1959.0200

[99] J.-X. Cheng, A. Volkmer, and X. S. Xie, “Theoretical and experimental characterization of coherent anti-Stokes Raman scattering microscopy,” J. Opt. Soc. Am. B 19, 1363–1375 (2002). 10.1364/josab.19.001363

[100] G. M. Hale and M. R. Querry, “Optical constants of water in the 200-nm to 200-*μ*m wavelength region,” Appl. Opt. 12, 555–563 (1973). 10.1364/ao.12.000555 20125343

[101] S. L. Jacques, “Optical properties of biological tissues: A review,” Phys. Med. Biol. 58, R37–R61 (2013). 10.1088/0031-9155/58/11/r37 23666068

[102] P. Ebersbach, N. Smirnoff, C. H. Camp, Jr, and J. Moger, “Chemical fingerprint imaging in planta with broadband coherent anti-Stokes Raman scattering microscopy,” Anal. Chem. 97, 16868–16876 (2025). 10.1021/acs.analchem.5c01980 40721985 PMC12355479

